# A single center observational study on emergency department clinician non-adherence to clinical practice guidelines for treatment of uncomplicated urinary tract infections

**DOI:** 10.1186/s12879-016-1972-6

**Published:** 2016-11-04

**Authors:** Catherine Zatorski, Mark Zocchi, Sara E. Cosgrove, Cynthia Rand, Gillian Brooks, Larissa May

**Affiliations:** 1 Department of Emergency Medicine, The George Washington University, 2120 L Street, NW Suite 4-450, Washington, DC, 20037 USA; 2 Center for Healthcare Innovation & Policy Research, The George Washington University, 2100 Pennsylvania Avenue Suite 300, Washington, DC 20037 USA; 3 Department of Medicine, Division of Infectious Diseases, Johns Hopkins Medical Institutions, Osler 425, 600 N. Wolfe St., Baltimore, MD 21287 USA; 4 Division of Pulmonary and Critical Care Medicine, The Johns Hopkins Institutions, 5501 Hopkins Bayview Circle, Baltimore, MD 21224 USA; 5 Department of Emergency Medicine, UC Davis Medical Center, 4150 V Street, Suite 2100, Sacramento, CA 95817 USA

**Keywords:** Antimicrobial stewardship, Urinary tract infection (UTI), Broad-spectrum antibiotics, Cystitis, Pyelonephritis

## Abstract

**Background:**

The Emergency Department (ED) is a frequent site of antibiotic use; poor adherence with evidence-based guidelines and broad-spectrum antibiotic overuse is common. Our objective was to determine rates and predictors of inappropriate antimicrobial use in patients with uncomplicated urinary tract infections (UTI) compared to the 2010 International Clinical Practice Guidelines (ICPG).

**Methods:**

A single center, prospective, observational study of patients with uncomplicated UTI presenting to an urban ED between September 2012 and February 2014 that examined ED physician adherence to ICPG when treating uncomplicated UTIs. Clinician-directed antibiotic treatment was compared to the ICPG using a standardized case definition for non-adherence. Binomial confidence intervals and student’s t-tests were performed to evaluate differences in demographic characteristics and management between patients with pyelonephritis versus cystitis. Regression models were used to analyze the significance of various predictors to non-adherent treatment.

**Results:**

103 cases met the inclusion and exclusion criteria, with 63.1 % receiving non-adherent treatment, most commonly use of a fluoroquinolone (FQ) in cases with cystitis (97.6 %). In cases with pyelonephritis, inappropriate antibiotic choice (39.1 %) and no initial IV antibiotic for pyelonephritis (39.1 %) where recommended were the most common characterizations of non-adherence. Overall, cases of cystitis were no more/less likely to receive non-adherent treatment than cases of pyelonephritis (OR 0.9, 95 % confidence interval 0.4–2.2, *P* = 0.90). In multivariable analysis, patients more likely to receive non-adherent treatment included those without a recent history of a UTI (OR 3.8, 95 % CI 1.3–11.4, *P* = 0.02) and cystitis cases with back or abdominal pain only (OR 11.4, 95 % CI 2.1–63.0, *P* = 0.01).

**Conclusions:**

Patients with cystitis with back or abdominal pain only were most likely to receive non-adherent treatment, potentially suggesting diagnostic inaccuracy. Physician education on evidence-based guidelines regarding the treatment of uncomplicated UTI will decrease broad-spectrum use and drug resistance in uropathogens.

## Background

The burden of increasingly resistant bacterial infections strains our healthcare system and is a major global public health challenge [1]. Antimicrobial stewardship programs aim to improve antimicrobial use and reduce emergence of resistance through a variety of mechanisms, but few systematic efforts to date have been made to perform antimicrobial stewardship in the emergency department (ED) setting [2]. The ED is a frequent site of antibiotic use and thus, provides a unique opportunity for antimicrobial stewardship. Up to half of all antimicrobial prescriptions are inappropriate [3], with poor adherence with evidence based guidelines (EBG) for infectious diseases [4, 5] and overuse of broad spectrum antibiotics [6, 7]; however, this has not been quantified in an ED setting.

Urinary tract infections (UTI) are one of the most common complaints diagnosed in the ED and accounted for approximately 2.4 million visits in 2010 [8]. Uncomplicated UTIs encompass acute, uncomplicated cystitis and pyelonephritis in outpatient, non-pregnant women [9]. Given that nearly half of all ED visits are by patients with non-life-threatening problems [10], emergency physicians must be adept at treating these illnesses. However, the natural limitations of the ED setting, such as inadequate microbiological testing and follow up, necessitates the use of treatment recommendations based on clinical findings in EBGs to minimize antimicrobial resistance and adverse events associated with broad spectrum antibiotic use.

The 2010 International Clinical Practice Guidelines (ICPG) established by the Infectious Diseases Society of America (IDSA) and the European Society for Microbiology and Infectious Diseases (ESCMID) updated the previous 1999 recommendations to account for changes in resistance, the increased adverse effects of antimicrobial therapy, and new antimicrobial agents available. It remains the most current guideline regarding treatment of uncomplicated UTI endorsed by the IDSA. In the absence of fever, flank pain, or other symptoms of pyelonephritis, fluoroquinolones are no longer first-line due to the increase in resistance and the propensity for side effects, a major departure from the 1999 guidelines [11]. Nitrofurantoin, fosfomycin, and trimethoprim-sulfamethoxazole (TMP-SMX) where community resistance rates are <20 % are recommended as first-line therapy for cystitis. Ciprofloxacin is preferred for pyelonephritis [9].

Our study objective was to determine proportions and predictors of inappropriate antimicrobial use as defined by 2010 ICPG established by the IDSA and the ESMID in emergency department patients presenting with a urinary tract infection.

## Methods

This was a prospective, observational study that was reviewed and approved by the Institutional Review Board at The George Washington University. We enrolled non-pregnant women, between the ages of 18 and 49 years, in an urban, academic ED in a tertiary care hospital in Washington, DC, USA with approximately 75,000 patient visits per year between September 2012 and February 2014. All participants provided informed consent in accordance with standards set forth by HHS regulations 45 CFR 46.11a and FDA regulations 21 CFR 50.25. IDSA guidelines are endorsed by hospital infection control for antibiotic choice and a hospital-wide antibiogram is not readily available to ED clinicians and is not specific to uropathogens. Inclusion criteria included complaints of symptoms consistent with a UTI, including dysuria, increased frequency of urination, and hesitancy to urinate, those who had a urine dipstick, urinalysis with microscopy, and/or urine culture ordered. Results of the urine culture were not available to the clinical staff during the course of patient care and were used exclusively for research purposes or clinical follow up. Women were excluded if they were previously treated for a UTI within the past month due to the possibly of treatment failure from the previous infection [12–14], were currently on antibiotics, had severe co-morbidities (diabetes mellitus, end stage renal disease, or immunocompromised) or had a recent urologic procedure or indwelling catheter. Enrollment occurred Monday to Friday, 8 am–7 pm, and Saturday to Sunday, 9 am–5 pm, due to the presence of department research assistants. Consecutive enrollment was attempted during this time. Clinical decision-making was at the discretion of the individual healthcare provider. At our site care for uncomplicated UTIs is primarily performed in the ED ambulatory care track by emergency medicine (EM) trained physicians and physician assistants (PA); however, EM residents (Post Graduate Years 1 through 4) and non-EM residents who rotate in the ED and also provide care. All clinical decisions made by physician assistants and EM residents are reviewed and approved by a board-certified EM physician prior to patient discharge. Pharmacists do not approve antibiotic prescriptions prior to administration or discharge.

Potential participants were screened for eligibility using the ED’s electronic medical record (EMR) and, if eligible, were approached for participation. Those who gave informed consent had demographic, behavioral, clinical, and treatment information collected through structured interview with the patient and clinician. Treatment information was collected by interview right after the clinical encounter. Urine cultures were prospectively collected to ascertain pathogen type and susceptibility information. Urine dipstick, urinalysis with microscopy, and culture results were collected via chart abstraction of the EMR.

Clinician-directed antibiotic treatment was compared to the guideline recommendations, and took into account allergy. No attempt was made to familiarize treating providers with guideline recommendations. We developed a standard case definition for non-adherence. Antibiotic use was not considered consistent with guidelines if the patient received a FQ in the absence of fever and/or flank pain and did not have a documented allergy to nitrofurantoin or to sulfonamides, received less than the recommended dose or duration according to the IDSA guidelines, or had a regimen longer than 2 days over the recommended duration. Clinical and demographic characteristics were collected via structured data form and chart abstraction was performed for verification. Given our institution does not have an outpatient or ED specific antibiogram and the hospital wide antibiogram is not widely distributed in this setting, we assumed the use of TMP-SMX as guideline adherent since providers may assume lower resistance for patients with uncomplicated infection. Final International Classification of Disease 9 (ICD9) diagnosis was reviewed by an independent emergency physician to ensure that diagnosis correlated with signs and symptoms recorded in the patient’s electronic medical record. We categorized cases as pyelonephritis or cystitis through direct interview of the clinician as well as review of the electronic medical record of the patient’s symptoms and the treating provider’s clinical impression as documented on the chart. Our case definition of pyelonephritis included the presence of fever or flank pain as per patient report. In instances where our case definitions did not match the clinical diagnosis we evaluated adherence per the clinician diagnosis (*n* = 11). We conduct a sensitivity analysis with these cases excluded and compare our results.

Results were summarized using proportions (%) for categorical data with binomial confidence intervals. Categorical variables were compared using either Pearson’s Chi-squared or Fisher’s exact test, where appropriate. To determine the predictors of non-adherence, univariate followed by multivariable logistic regression analysis were performed. Predictors included: patients’ age, race (black, white, or other), provider type (resident vs. attending or PA), current sexual activity (yes/no), prior history of UTI outside the 1-month exclusion, diagnosis of pyelonephritis, decreased urine output, frequent urination, dysuria, abdominal pain, back pain, nausea/vomiting, vaginal discharge, days since onset of symptoms and urine analysis result. All variable was considered potentially significant and further analyzed in a stepwise multivariate logistic regression model using the backward selection method for determining significant independent factors at *P* < 0.2. While these are a large number of variables to include, prior research demonstrates a variety of factors leading to overprescribing of broad spectrum antibiotics in cases of diagnostic uncertainty or clinical presentation, thus we felt it important to include demographic, clinical and test factors in our model [15, 16]. To reduce the number of variables included in the regression analyses, we collapsed urinary symptoms together (dysuria, frequent urination, and decreased urination) and non-urinary clinical symptoms together (back pain, abdominal pain, nausea/vomiting, and vaginal discharge). Odds ratios with 95 % confidence intervals were calculated. Predictors with a *p*-value of less than 0.05 were considered statistically significant. The final model was tested for fit using a Hosmer-Lemeshow *X* ^2^ (1.84, *P* = 0.97). Stata, v.13 (College Station, TX, USA) was used for all analyses.

## Results

A total of 103 of 445 patients met the inclusion and exclusion criteria. Of these, a total of 65 (63.1 %) received treatment that did not adhere to the ICPG guidelines. Sixty-seven patients were identified as cystitis, of which 42 received treatment non-adherent to the guidelines (62.7 %). The most common characterization of non-adherence in cystitis was inappropriate use of a FQ, which occurred in 97.6 % (41/42) of the non-adherent cases. Thirty-six patients were identified as Pyelonephritis, of which 23 received treatment non-adherent to the guidelines (63.9 %). The most common description of non-adherence in pyelonephritis was inappropriate antibiotic choice and no initial IV antibiotic, which both occurred in 39.1 % (9/23) of the non-adherent cases. We found that 22.2 % of *E. coli* isolates from patients with uncomplicated UTI in our study were resistant to TMP-SMX, and 8.0 % to FQ (data not shown).

Baseline characteristics of patients with pyelonephritis compared to cystitis are presented in Table 1. Patients with pyelonephritis had higher prevalence of abdominal pain (75.0 % vs. 43.3 %, *P* < 0.01), back pain (69.4 % vs. 28.4 %, *P* < 0.01), nausea/vomiting (36.1 % vs 11.9 %, *P* < 0.01) than patients with cystitis. They were also more likely to have had symptoms for more than 4 days (58.3 vs 28.8 %, *P* < 0.01).

**Table 1:** Patient demographic and clinical characteristics of 103 adults treated for uncomplicated UTI

Characteristics	Overall (*n* = 103)		Pyelonephritis (*n* = 36)		Cystitis (*n* = 67)		*P*-value
	%	(95 % CI)	%	(95 % CI)	%	95 % CI)	
Race/ethnicity							
Black, non-Hispanic	51.5	(41.4–61.4)	55.6	(38.1–72.1)	49.3	(36.8–61.8)	0.74
White, non-Hispanic	31.1	(22.3–40.9)	30.6	(16.3–48.1)	31.3	(20.6–43.8)	
Hispanic or other race	17.5	(10.7–26.2)	13.9	(4.7–29.5)	19.4	(10.8–30.9)	
Age, y							
18–29	64.1	(54.0–73.3)	47.2	(30.4–64.5)	73.1	(60.9–83.2)	**<0.01**
30–39	25.2	(17.2–34.8)	44.4	(27.9–61.9)	14.9	(7.4–25.7)	
40+	10.7	(5.5–18.3)	8.3	(1.8–22.5)	11.9	(5.3–22.2)	
Clinical history							
Sexually active	83.5	(74.9–90.1)	77.8	(60.8–89.9)	86.6	(76.0–93.7)	0.25
No recent history of UTI	38.8	(29.4–48.9)	36.1	(20.8–53.8)	40.3	(28.5–53.0)	0.68
Clinical symptoms							
Dysuria	78.6	(69.5–86.1)	75.0	(57.8–87.9)	80.6	(87.5–99.1)	0.51
Frequent urination	83.5	(74.9–90.1)	83.3	(67.2–93.6)	83.6	(72.5–91.5)	0.97
Decreased urine output	37.9	(28.5–48.0)	47.2	(30.4–64.5)	32.8	(21.8–45.4)	0.15
Nausea/vomiting	20.4	(13.1–29.5)	36.1	(20.8–53.8)	11.9	(5.3–22.2)	**<0.01**
Vaginal discharge	13.6	(7.6–21.8)	11.1	(3.1–26.1)	14.9	(7.4–25.7)	0.59
Abdominal pain	54.4	(44.3–64.2)	75.0	(57.8–87.9)	43.3	(31.2–56.0)	**<0.01**
Back pain	42.7	(33.0–52.8)	69.4	(51.9–83.7)	28.4	(18.0–40.7)	**<0.01**
Onset of symptoms							
< 4 days	60.8	(50.6–70.3)	41.7	(25.5–59.2)	71.2	(58.7–81.7)	**0.01**
4–7 days	29.4	(20.8–39.3)	41.7	(25.5–59.2)	22.7	(13.3–34.7)	
> 7 days	9.8	(4.8–17.3)	16.7	(6.4–32.8)	6.1	(1.7–14.8)	

*P*-Values that appear in **bold** are significant at *p* < 0.05

Characteristics of patient management and diagnostic results of patients with pyelonephritis versus cystitis are shown in Table 2. Residents were more likely to treat patients with pyelonephritis compared to cystitis (52.8 vs 25.4 %; *P* < 0.01). Non-adherence to guidelines was similar for patients with pyelonephritis and cystitis (63.9 % vs. 62.7 %; *P* = 0.90).

**Table 2:** Clinical treatment, test results, and adherence to IDSA guidelines for 103 adults with uncomplicated UTI

Characteristics	Overall (*n* = 103)		Pyelonephritis (*n* = 36)		Cystitis (*n* = 67)		*P*-value
	%	(95 % CI)	%	(95 % CI)	%	(95 % CI)	
Provider							
Attending or PA	65.0	(55–74.2)	47.2	(30.4–64.5)	74.6	(62.5–84.5)	0.01
Resident	35.0	(25.8–45)	52.8	(35.5–69.6)	25.4	(15.5–37.5)	
Antibiotics prescribed							
Ciprofloxacin	59.2	(49.1–68.8)	47.2	(30.4–64.5)	65.7	(53.1–76.8)	0.09
Nitrofurantoin	20.4	(13.1–29.5)	22.2	(10.1–39.2)	19.4	(10.8–30.9)	
TMP-SMX	18.4	(11.5–27.3)	25.0	(12.1–42.2)	14.9	(7.4–25.7)	
Other	1.9	(0.2–6.8)	5.6	0.7–18.7)	0.0	(0.0–5.4)	
Urinalysis results							
Positive	76.7	(67.3–84.5)	72.2	(54.8–85.8)	79.1	(67.4–88.1)	0.43
Negative	23.3	(15.5–32.7)	27.8	(14.2–45.2)	20.9	(11.9–32.6)	
Culture results ^a^							
Escherichia coli	60.6	(42.1–77.1)	50.0	(24.7–75.3)	70.6	(44–89.7)	0.23
Other pathogen	39.4	(22.9–57.9)	50.0	(24.7–75.3)	29.4	(10.3–56)	
Non-adherence to guidelines	63.1	(53.0–72.4)	63.9	(46.2–79.2)	62.7	(50–74.2)	0.90

^a^ Only includes cultures that had pathogen growth (*n* = 42)

In the univariate analysis, patients with cystitis with back or abdominal pain had increased odds of receiving non-adherent treatment compared to patients with cystitis but no other non-urinary symptoms (OR 8.8, 95 % CI 1.7–45.9) as were patients with no recent history of an UTI (OR 2.4, 95 % CI 1.0–5.7) (Table 3). Patients with nausea or vomiting symptoms (any diagnosis) had reduced odds of receive guideline non-adherent treatment compared to patients without those symptoms (OR 0.3; 95 % CI 0.1–0.9). The multivariable model included, no recent history of UTI, type of UTI with symptoms, and nausea/vomiting. (Table 4). Physicians were more likely to be guideline non-adherent for patients with no recent history of UTI (OR 3.8; 95 % CI 1.3–11.4) –0.9), had cystitis with back or abdominal symptoms only (OR 11.4; 95 % CI 2.1–63.0), or had cystitis with vaginal discharge (OR 12.1; 95%CI 1.1–137.5). The wide confidence intervals of the clinical subgroups with small numbers of patients should be interpreted with caution. In a sensitivity analysis excluding patients where the clinician diagnosis did not match our case definition (*n* = 11) nausea/vomiting was dropped from the model and cystitis with vaginal discharge was no longer a significant predictor of non-adherence (*p* = 0.07). Cystitis with back or abdominal pain only and no recent history of UTI remained significant.

**Table 3:** Univariate logistic regression of non-adherence to antibiotic guidelines for uncomplicated UTI

Variable	Non-Adherent %	OR	95 % CI	*P*-value
Race				
Black, non-Hispanic	66.0	1		
White, non-Hispanic	59.4	0.8	0.3–1.9	0.54
Hispanic or other race	61.1	0.8	0.3–2.4	0.71
Age				
18–29	63.6	1		
30–39	61.5	0.9	0.4–2.3	0.85
40+	63.6	1.0	0.3–3.8	1.00
Clinical history				
Sexually active	62.8	0.9	0.3–2.7	0.88
No recent history of UTI	75.0	2.4	1.0–5.7	**0.05**
Onset of symptoms				
< 4 days	67.7	1		
4–7 days	56.7	0.6	0.3–1.5	0.30
> 7 days	60.0	0.7	0.2–2.8	0.63
Provider				
Attending or PA	64.2	1		
Resident	61.1	0.9	0.4–2.0	0.76
Clinical symptoms				
Dysuria	64.2	1.2	0.5–3.3	0.66
Frequent urination	61.6	0.7	0.2–2.1	0.49
Decreased urination	61.5	0.9	0.4–2.0	0.80
Nausea/vomiting	42.9	0.3	0.1–0.9	**0.04**
Vaginal discharge	85.7	4.1	0.9–19.3	0.08
Back pain	61.4	0.9	0.4–2.0	0.75
Abdominal pain	62.5	0.9	0.4–2.1	0.90
Type of UTI				
Pyelonephritis	62.8	1		
Cystitis	55.6	0.9	0.4–2.2	0.90
Type of UTI with clinical symptom combinations				
Cystitis, no other non-urinary symptoms	52.0	1		
Cystitis, with back or adnominal pain symptoms only	90.5	8.8	1.7–45.9	**0.01**
Cystitis, with vaginal discharge	90.0	8.3	0.9–75.7	0.06
Cystitis, with other combinations of non-urinary symptoms	9.1	0.1	0.0–0.8	**0.03**
Pyelonephritis (any fever or flank pain present)	63.9	1.6	0.6–4.6	0.36
Urinalysis results				
Positive	58.2	1		
Negative	79.2	2.7	0.9–8.0	0.07

*P*-Values that appear in **bold** are significant at *p* < 0.05

Non-urinary symptoms include: back pain, abdominal pain, fever, flank pain, vaginal discharge, or nausea/vomiting

**Table 4:** Multivariable logistic regression analysis of non-adherence to antibiotic guidelines for uncomplicated UTI

Variable	OR	95 % CI	*P*-value
Clinical history			
No recent history of UTI	3.8	1.3–11.4	**0.02**
Clinical symptoms			
Nausea/vomiting	0.4	0.1–1.4	0.14
Type of UTI, symptoms			
Cystitis, no other non-urinary symptoms	1		
Cystitis, with back or adnominal pain symptoms only	11.4	2.1–63.0	**0.01**
Cystitis, with vaginal discharge	12.1	1.1–137.5	**0.05**
Cystitis, with other combinations of non-urinary symptoms	0.1	0.0–1.1	0.06
Pyelonephritis (fever or flank pain present)	2.7	0.8–9.3	0.12

*P*-Values that appear in **bold** are significant at *p* < 0.05

Non-urinary symptoms include: back pain, abdominal pain, fever, flank pain, vaginal discharge, or nausea/vomiting

## Discussion

We found a high degree of non-adherence to current guidelines for the treatment of UTI; namely, the overuse of FQ in patients with uncomplicated cystitis and inappropriate antibiotic choice and no initial IV antibiotic for pyelonephritis. Interestingly, providers were more likely to be non-adherent with guidelines for cystitis cases where patients only had back or abdominal pain and for cases without a recent history of UTI. Providers were also more likely to be guideline non-adherent for patients with vaginal discharge, however this result was not statistically significant in our sensitivity analysis. The results potentially suggest diagnostic inaccuracy as well as overuse of broad spectrum antibiotics for patients with vaginal discharge and prior UTI. Only 19.4 % of our patients with uncomplicated cystitis received the first line agent of nitrofurantoin, despite the low rate of resistance compared to other antimicrobial agents. Reasons for this might include the perception that FQ are more effective, or the shorter treatment duration of FQ, which providers may perceive as more convenient for patients. Furthermore, 22.2 % of patients with symptoms of pyelonephritis received this medication, despite recommendations not to use nitrofurantoin for patients with pyelonephritis. Other studies have documented inappropriate antibiotic use for common infections in the ED and other settings [2, 3]. It is also possible that our structured interview detected symptoms consistent with pyelonephritis whereas the clinician was basing their assessment on different criteria, including physical examination. It is nonetheless concerning that there is a potential for misdiagnosis of these cases.

While UTIs are a common diagnosis and account for more than 2 million patient visits per year in hospital based settings alone, overall adherence to treatment guidelines has been historically low. In a 2011 cross-sectional study of lower UTIs in a general practice clinic (*n* = 658) performed over a 3-month period, researchers found that antibiotic treatment was given in 634 (96.4 %) cases and only 92 (17.7 %) of these received first line antibiotics [17].

Although definitive diagnosis of cystitis is made with clinical symptoms and bacteriuria, dysuria, frequency, and urgency have all been associated with the diagnosis of UTI [17, 18]. While the urine dipstick may be helpful in diagnosing UTI, it is unable to effectively rule it out with moderate clinical suspicion. Positive leukocyte esterase or nitrate has been found to have a positive likelihood ratio of 2.5 for the diagnosis of UTI [19]. We found that a negative urinalysis increased the odds of non-adherence by more than 2-fold, although this was not statistically significant. This may be because patients with atypical features or negative results were treated with antibiotics for other conditions, or because clinicians were more likely to use broad spectrum antibiotics, such as FQ, for an uncertain diagnosis.

There were several limitations to our study. First, we enrolled participants at a single urban academic center using a convenience sample, which may not be representative of other sites. Given the small number of physicians sampled, individual errors or differing practice styles can influence the study greatly. Nonetheless, we found similar proportions of FQ use compared to other studies in and outside the ED setting [15, 20]. Furthermore, while proportions of antibiotic prescribing could potentially vary between academic and community based centers, we found no significant prescribing differences between attending physicians and residents. In addition, we did not directly ascertain providers’ reasoning regarding antibiotic selection, which may have yielded additional insight into empiric antibiotic selection, such as coverage for other suspected etiologies. While we used a clinical diagnosis rather than microbiologic culture to confirm the diagnosis of UTI, we think our strategy of reviewing charts for inclusion of patients with uncomplicated cystitis and pyelonephritis based on clinical signs and symptoms as well as change prescribing practices documented diagnosis is appropriate given recommendations for empiric treatment without culture in most cases with uncomplicated infection [9]. The inclusion of pyelonephritis secondary to untreated cystitis or resistant pathogen was minimized by not enrolling women who were treated for urinary complaints within 1 month of presentation. However, we cannot eliminate it as a possibility and thus it represents an important limitation to our study. We also recognize the small sample size may have limited our ability to identify significant predictors of non-adherence. Odds ratios for clinical symptoms have wide confidence intervals and should be interpreted with caution. While we assumed adherence with TMP-SMX, microbiologic results show that > 20 % of isolates from patients with uncomplicated UTI are resistant to TMP-SMX, however this was not known to the treating providers during the study enrollment period. Finally, we cannot know whether providers made antibiotic treatment decisions based on prior microbiologic culture.

There were several cases where the clinician diagnosis differed from our case definition. For example, in seven cases the clinician diagnosis was cystitis in patients with documented fever or flank pain and in four cases the clinician diagnosis was pyelonephritis without documented fever or flank pain. While it is possible that clinicians based their diagnosis of cystitis versus pyelonephritis based on physical examination findings, this calls to question whether patients with symptoms consistent with pyelonephritis such as flank pain and fever, may be undertreated, and warrants further investigation.

Finally, we did not distribute guidelines surrounding UTI to our clinicians, but we cannot be certain that awareness of the study did not change prescribing practices to be more adherent to the recommended guidelines.

The ED is a unique care setting where physicians must make quick treatment decisions about prescribing antibiotics for UTIs with often incomplete information and limited opportunity for follow up. A previous study conducted by our group showed that clinical decision making regarding antibiotics for patients is complex and is affected by limited environmental resources, lack of access to decision making tools including locally relevant guidelines, clinical inertia, and perceptions that broad spectrum antibiotics are superior in cases where follow up is not feasible [17]. In the case of uncomplicated UTI, however, this overuse of broad-spectrum antibiotics is in fact counterproductive, given the increasing resistance rates to FQ.

A primary cause of provider non-adherence is a lack of knowledge of antimicrobial guidelines. A 2000 cross-disciplinary survey of 92 delegates with an interest in UTIs showed that <3 % answered correctly [21]. While guidelines can be useful to guiding prescribing [22], many ED providers are skeptical as they feel the ED is a special population and that guidelines should be locally tailored. Lack of knowledge surrounding guidelines is also common [21]. Strategies to implement ED interventions in the form of validated clinical decision rules have yielded mixed results [23, 24]. However, there are unique facilitators to successful practice change in the ED, including interest in novel tools and acceptance of new responsibilities [26]. Interventions to address barriers to change in the ED include educational outreach, formal feedback to the clinical care team, and process change [25]. Best practices solutions utilize a multifaceted approach [27, 28]. Novel strategies include embedding guidelines in the electronic health record [29]. A recent study found that a stewardship intervention that included an electronic order set with audit and feedback found that adherence to UTI guidelines increased from 44 % at baseline to 82 % while use of FQ for uncomplicated cystitis decreased from 44 to 13 % overall [30]. Given the frequency of visits for uncomplicated UTI in the ED, and the importance of FQ in contributing to increasing trends in resistance, efforts should focus specifically on the use of FQ in the ED for patients with uncomplicated infections. However, it remains uncertain if these effects can be sustained over time and further studies are urgently needed to help reduce inappropriate broad-spectrum antibiotic use and improve adherence to clinical practice guidelines in patients with uncomplicated UTI and other infections.

## Conclusions

63.1 % of patients who were treated in the ED for uncomplicated UTI received antibiotics that did not adhere to ICPG. The most common characterization of non-adherence was use of FQ in cystitis, followed by an inappropriate duration, no initial IV antibiotic for pyelonephritis, and another inappropriate antibiotic choice. Interestingly, physicians were less likely to be guideline non-adherent when patient’s reported back or abdominal pain.

## Abbreviations

EBG: Evidence based guidelines
ED: Emergency department
EM: Emergency medicine
ESCMID: European Society of Microbiology and Infectious Diseases
FQ: Fluoroquinolone
ICPG: International Clinical Practice Guidelines
IDSA: Infectious Disease Society of America
PA: Physician assistant
STI: Sexually Transmitted Infection
TMP-SMX: Trimethoprim-sulfamethoxazole
UTI: Urinary tract infections
